# Eating disorders in sexual minority adolescents and young adults: examining clinical characteristics and psychiatric co-morbidities in an inpatient medical setting

**DOI:** 10.1186/s40337-023-00756-5

**Published:** 2023-02-28

**Authors:** Anita V. Chaphekar, Amanda Downey, Andrea K. Garber, Mikayla Kuykendall, Paola Bojorquez-Ramirez, Kyle T. Ganson, Sara M. Buckelew, Jason M. Nagata

**Affiliations:** 1 Department of Pediatrics, University of California, San Francisco, 550 16th Street, Box 0503, San Francisco, CA 94143 USA; 2 Department of Psychiatry and Behavioral Sciences, University of California, San Francisco, 550 16th Street, Box 0503, San Francisco, CA 94143 USA; 3 Nutrition and Food Services, San Francisco Medical Center, University of California, 1855 Fourth St, San Francisco, CA 94143 USA; 4 Yale School of Public Health, Yale University, 60 College St, New Haven, CT 06510 USA; 5 Factor-Inwentash Faculty of Social Work, University of Toronto, 246 Bloor St W, Toronto, ON Canada

**Keywords:** Sexual minority individuals, Gay, Lesbian, Bisexual, Eating disorders, Inpatient medical unit

## Abstract

**Background:**

Sexual minority adolescents and young adults are at higher risk of eating disorders compared to heterosexual peers. However, little is known about the clinical and psychiatric presentation of this population requiring inpatient medical stabilization. Given the increased risk for eating disorder behaviors in sexual minority individuals amidst increased rates of medical hospitalizations secondary to eating disorders, it is important to understand presenting characteristics of this population. The objectives of this study were to (1) describe the clinical characteristics of sexual minority adolescents and young adults with eating disorders admitted for medical instability and (2) compare psychiatric co-morbidities and suicidality of sexual minority adolescents and young adults to heterosexual peers.

**Methods:**

A retrospective chart review was conducted of 601 patients admitted to a large inpatient eating disorders medical stabilization unit between 2012 and 2020. Data collected included demographics, medical data including vital signs, and psychiatric characteristics. Chi square or t-tests were used to examine potential differences in clinical characteristics and psychiatric co-morbidities between groups. Modified Poisson regression was used to assess associations between sexual orientation and psychiatric co-morbidities.

**Results:**

Over one fifth (21.1%, n = 103) of our inpatient sample identified as a sexual minority individual. The average age of participants was 15.6 years (2.7). Sexual minority adolescents and young adults had higher percent median body mass index compared to heterosexual peers and yet equally severe vital sign instability on admission. Sexual minority adolescents and young adults were almost 1.5 times more likely to have a psychiatric comorbidity with higher rates of depression, anxiety, and post-traumatic stress disorder. Sexual minority adolescents and young adults were approximately two times more likely to have a history of self-injurious behaviors and/or suicidality.

**Conclusions:**

Sexual minority adolescents and young adults with eating disorders have equally severe vital sign instability despite higher percent median body mass index on admission for medical stabilization. Sexual minority adolescents and young adults hospitalized for medical complications of eating disorders are far more likely to have an additional mental health disorder and a history of self-harm and/or suicidality, which may portend a less favorable long-term prognosis.

## Plain English summary

Eating disorders are highly prevalent among sexual minority individuals, including those who identify as gay, lesbian, or bisexual. Little is known about the medical and psychiatric profile of sexual minority adolescents and young adults with eating disorders who require inpatient hospitalization for medical stabilization. This study examines the clinical characteristics and psychiatric comorbidities of sexual minority adolescents and young adults compared to heterosexual peers hospitalized for medical complications of eating disorders from 2012-2020. The average age of our participants was 15 years old. The majority of participants were assigned female at birth and were White/Caucasian. We found that sexual minority adolescents and young adults were just as medically compromised as heterosexual peers and more likely to have a co-morbid diagnosis of depression, anxiety, and/ or post-traumatic stress disorder. Additionally, sexual minority individuals were more likely to have a history of suicidal ideation and self-injurious behavior. These findings may have important implications for long-term prognosis and potential for recovery.

## Background

Despite advances in nutritional rehabilitation and evidence-based psychological interventions in the treatment of eating disorders, the incidence of eating disorders in adolescents and young adults (AYA) continues to rise [1, 2]. Dangerously low body weight, hemodynamic instability, and electrolyte abnormalities secondary to an eating disorder can yield serious medical complications requiring hospitalization; however, there is a paucity of research on these medical complications in sexual minority populations with eating disorders [3]. Individuals identifying as gay, lesbian, and bisexual are at increased risk for eating disorders and disordered eating behaviors [4, 5]. Studies have reported up to 4 times higher odds of sexual minority individuals experiencing eating disorders in their lifetime and 1.5 times higher odds of having disordered eating behaviors [4–6]. While the majority of this research has been in the adult population, studies have shown an increased prevalence of eating disorder behaviors such as fasting (24.8%) and purging (16–17%) in sexual minority adolescents compared to heterosexual peers [5, 7].

AYA with eating disorders are highly likely to have an additional co-morbid psychiatric disorder [2]. More than half of individuals with anorexia nervosa (AN) and bulimia nervosa (BN) have a comorbid psychiatric disorder [2]. While AN and BN have different comorbidity profiles, depression, anxiety, post-traumatic stress disorder (PTSD) and attention deficit hyperactivity disorder (ADHD) are highly prevalent among all eating disorder diagnoses [2, 8, 9]. Additionally, sexual minority individuals, including children and adolescents, experience higher rates of mental health conditions compared to heterosexual individuals [10–12]. One study examining depressive symptoms among high school students across the United States reported a prevalence of 60.4% in sexual minority adolescents compared to 26.4% in heterosexual peers [11]. Presence of a comorbid psychiatric disorder portends negative long-term outcomes for patients with eating disorders [2, 8, 9, 13, 14]. This is of utmost concern in sexual minority adolescents and young adults (SM AYA), a population which already has higher rates of preoccupation with body weight and appearance, weight control behaviors, and mental health conditions compared to heterosexual peers [10, 15–23].

Elevated rates of self-injurious and suicidal behaviors are common, with suicide now being the second leading cause of mortality among patients with AN [24]. Approximately half of adolescent patients with AN struggle with suicidal ideation and more than one-third of adolescents with BN have attempted suicide [2]. Specifically, in the SM AYA population, studies report sexual orientation as a risk factor for suicide attempts and describe SM individuals as being at a higher risk for suicide attempt [20]. As SM AYA are more likely to have a history of suicide attempt and experience more suicidal thoughts and behaviors as compared to heterosexual peers, SM AYA with eating disorders may be especially vulnerable to mental health comorbidities and unfavorable long-term outcomes.

To our knowledge, studies have not examined the clinical and psychiatric characteristics of SM AYA with eating disorders requiring inpatient medical stabilization. As hospitalization rates for eating disorders are on the rise and SM AYA have an increased risk for both eating disorders and mental health conditions, it is imperative to study this population to identify significant characteristics which could inform future care. This exploratory study aims to describe the clinical characteristics of SM AYA. We will also compare psychiatric co-morbidities and suicidality of SM AYA to their heterosexual peers admitted for medical stabilization. Given the mental health disparities in sexual minority individuals described above, we hypothesize that higher rates of suicidality and comorbid mental health diagnoses will be observed among SM AYA compared to heterosexual peers in our inpatient sample.

## Methods

### Study design, participants, and study setting

A retrospective chart review was conducted of 601 adolescents and young adults, aged 9 to 25 years, admitted to a large inpatient medical stabilization unit at the University of California, San Francisco (UCSF) between May 2012 and August 2020. This timeframe was chosen as electronic data prior to May 2012 was not available. Patients were admitted for bradycardia, hypotension, orthostasis, rapid weight loss or extremely low body weight, and electrolyte abnormalities per Society of Adolescent Health and Medicine indications for supporting hospitalization in an adolescent with an eating disorder [3]. The goal of hospitalization is medical stabilization through nutritional rehabilitation. Refeeding and electrolyte monitoring protocols have been described in detail elsewhere [25]. Our inpatient program has an interdisciplinary team comprised of physicians, dietitians, psychologists, and social workers with eating disorder expertise that meet with each patient.

### Measurements

Data was collected as a part of a larger UCSF eating disorder medical registry including age, vital signs, height, weight, laboratory values, sex assigned at birth, sexual orientation, eating disorder diagnosis, co-morbid psychiatric diagnoses, and suicidality or self-injurious behavior. The Institutional Review Board of the University of California, San Francisco, has approved the use of the eating disorder medical registry. Height, weight, and laboratory evaluation were measured within 24 h of admission. Body mass index (BMI) and median BMI (mBMI) were calculated using height and weight [26]. Heart rate and blood pressure nadirs were collected during the entirety of hospitalization. Procedures for vital sign measures, along with the protocols for electrolyte monitoring and replacement and weight assessments have been previously published [25, 27]. Length of hospitalization was measured in days and determined by subtracting discharge date from admission date.

Sexual orientation data were self-reported as part of the physicians’ history and physical at time of hospital admission and/or in the electronic medical record under “Sexual Orientation and Gender Identity”. SM AYA were grouped as one category/variable and defined as “lesbian”, “gay”, “bisexual”, “queer”, “unsure/questioning”, “pansexual”, or “asexual”. This is consistent with other studies that include “unsure” in the sexual minority category [28]. Participants with stated sexual orientation as “straight” were included in the heterosexual group. For the purposes of this study, gender identity (cisgender, transgender, non-binary) was excluded from data collection and analysis.

Eating disorder diagnosis was classified into three categories: AN, other specified feeding and eating disorders (OSFED) which includes atypical anorexia nervosa, and other. ‘Other’ included avoidant restrictive food intake disorder (ARFID), BN, and unspecified feeding or eating disorder. These diagnoses were grouped together to allow for a sufficient sample for analysis. A psychologist or psychiatrist gave participants an eating disorder diagnosis following psychological evaluation during inpatient admission per Diagnostic and Statistical Manual of Mental Disorders, Fifth Edition (DSM-5) [29]. The study team reviewed charts and reclassified diagnoses per DSM-5 criteria for patients hospitalized prior to the release of DSM-5 in 2013. Specifically, participants' electronic medical records were reviewed and those with a diagnosis of eating disorder not otherwise specified, a diagnosis no longer present in DSM-5, were found to meet DSM-5 criteria for unspecified feeding and eating disorder (n = 2).

Psychiatric diagnoses for participants included self-reported pre-existing psychiatric diagnoses and psychiatric diagnoses made during hospitalization by psychologists. Specific diagnoses included depression, anxiety, obsessive compulsive disorder (OCD), PTSD, and ADHD. For the purposes of this study, our category of depression included the specific DSM-IV and DSM-5 diagnoses of major depressive disorder, depressive disorder not otherwise specified, and unspecified depression. The anxiety category included generalized anxiety disorder, social anxiety disorder, anxiety not otherwise specified, and unspecified anxiety. Depression and anxiety diagnoses were not reclassified if participants were diagnosed using DSM-IV criteria. History of suicidal ideation, history of suicide attempt, self-injurious behavior, passive suicidal ideation during hospitalization, and active suicidal ideation during hospitalization were collected from the participant’s history and physical note or from the psychological assessment during hospitalization.

### Statistical analyses

Participants with missing sexual orientation data or without a DSM-5 eating disorder diagnosis were excluded from the study (n = 138). The final analytic sample consisted of 463 patients. All three categories of eating disorder diagnosis (as described above) were included in analysis. Fisher’s exact, Chi square or t-tests were used to examine potential differences in clinical characteristics and psychiatric co-morbidities between SM and heterosexual AYA. Sexual orientation was dichotomized. Modified Poisson regression analyses were conducted and transformed to risk ratios to examine associations between sexual orientation and psychiatric co-morbidities [30]. Models were adjusted for age, sex assigned at birth, and eating disorder diagnosis. Analyses used Stata 17 (Stata Corp LP, College Station, TX).

## Results

### Demographics, length of hospitalization, and medical data

Of the 463 participants, 99 (21%) identified as a SM and 364 (79%) identified as heterosexual (Table 1). 388 were assigned female at birth and 75 were assigned male at birth. The mean age of participants was 15.6 years (2.7) and 63% were White or Caucasian (Table 1).

**Table 1:** Demographics, length of hospitalization, and medical characteristics by sexual orientation^a^

Characteristic	Sexual Orientation			
	Total (N = 463)^b^	Heterosexual Individuals (N = 364)^c^	Sexual Minority Individuals (N = 99)^c^	*p*-Value^d^
Age, years	15.60 ± 2.68	15.67 ± 2.54	15.34 ± 3.13	0.286
*Sex assigned at birth*				0.063
Female	388 (83.80)	299 (82.14)	89 (89.90)	
Male	75 (16.20)	65 (17.86)	10 (10.10)	
*Race, N (%)*				0.133
White or Caucasian	291 (62.85)	239 (65.66)	52 (52.53)	
Asian or NHOPI^e^	42 (9.07)	28 (7.69)	14 (14.14)	
Black or African American	13 (2.81)	10 (2.75)	3 (3.03)	
Other	99 (21.38)	74 (20.33)	25 (25.25)	
Unknown/Declined	18 (3.89)	13 (4.92)	5 (5.05)	
*Ethnicity, N (%)*				0.628
Hispanic	88 (19.01)	70 (19.23)	18 (18.18)	
Non-Hispanic	351 (75.81)	277 (76.10)	74 (74.75)	
Unknown/Declined	24 (5.18)	17 (4.67)	7 (7.07)	
Length of hospitalization stay (days)	9.93 ± 6.47	10.18 ± 6.90	9.01 ± 4.44	0.111
BMI kg/m^2^	17.83 ± 2.82	17.72 ± 2.77	18.21 ± 3.01	0.132
Percent median body mass index^f^	88.11 ± 13.81	87.31 + 13.26	91.09 + 15.38	**0.016**
*Vital signs*				
Pulse (beats per minute) nadir during hospitalization	45.73 ± 10.61	45.98 ± 10.66	44.82 ± 10.45	0.333
Systolic Pressure (mmHg) nadir during hospitalization	89.92 ± 9.63	83.74 ± 9.46	84.57 ± 10.27	
Diastolic Pressure (mmHg) nadir during hospitalization	45.47 ± 7.22	45.35 ± 7.15	45.89 ± 7.50	
*Electrolyte analysis at admission*^*g*^				
Sodium (135–145 mmol/L)	138.09 ± 10.64	138.12 ± 11.93	137.92 ± 2.46	0.860
Potassium (3.5–5.0 mmol/L)	3.88 ± 0.681	3.89 ± 0.036	3.84 ± 0.065	0.499
Magnesium (1.7–2.2 mg/dL)	2.13 ± 0.394	2.13 ± 0.434	2.15 ± 0.192	0.720
Phosphorous (2.9–5.0 mg/dL)	4.05 ± 2.60	4.08 ± 2.91	3.93 ± 0.609	0.612
*Other laboratory evaluation at admission*^*g*^				
Triglycerides (< 200 mg/dL)	46.56 ± 33.24	45.99 ± 34.23	48.65 ± 29.36	0.507
Cholesterol (< 170 mg/dL)	166.11 ± 41.36	166.04 ± 41.86	166.37 ± 39.65	0.948
Hemoglobin (12–17 g/dL)	12.96 ± 1.24	12.95 ± 1.25	13.02 ± 1.22	0.652
Hematocrit (36–52%)	38.36 ± 3.53	38.31 ± 3.57	38.59 ± 3.40	0.498
Creatinine (0.45–1.08 mg/dL)	0.78 ± 1.84	0.80 ± 2.07	0.71 ± 0.16	0.676
Blood urea nitrogen (7–21 mg/dL)	11.91 ± 6.44	11.79 ± 6.74	122.35 ± 6.44	0.453
Thyroid stimulating hormone (0.45–4.33 mIU/L)	1.97 ± 1.44	2.03 ± 1.47	1.72 ± 1.27	0.065
Free thyroxine (10–18 pmol/L)	12.00 ± 1.82	12.04 ± 1.77	11.84 ± 2.01	0.353
Aspartate transaminase (13–35 U/L)	28.84 ± 35.15	29.69 ± 39.06	25.65 ± 11.86	0.328
Alanine transaminase (8–24 U/L)	22.07 ± 28.87	21.94 ± 30.62	22.57 ± 21.32	0.852
Albumin (3.5–5.0 g/dL)	4.32 ± 0.42	4.30 ± 0.40	4.38 ± 0.48	0.122

Bold face indicates significant p value

^a^Table values are mean ± SD for continuous variables and n (column %) for categorical variables

^b^Total number of participants with eating disorder diagnosis and sexual orientation listed in chart

^c^Percentages may not sum to 100% due to rounding

^d^T-test was used for continuous variables. Chi Square or Fisher's exact test if n < 5 was used for categorical variables

^e^NHOPI = Native Hawaiian and Other Pacific Islanders

^f^50th percentile body mass index for age and sex

^g^Reference range includes ranges for males and females over and under the age of 18 years

The average body mass index (BMI) at time of admission of the participants was 17.8 kg/m^2^ (2.8) (Table 1). The average percent median BMI (%mBMI) of the participants was 88.11% (13.8) which is consistent with mild malnutrition [31]. SM individuals had a higher %mBMI on admission of 91% compared to their heterosexual peers who had %mBMI of 87% (*p* = 0.016). Heart rate and blood pressure nadirs did not differ between groups (Table 1). Laboratory evaluation was comparable between groups (Table 1). The average length of hospitalization between SM and heterosexual AYA did not differ (9.0 ± 4.4 days vs. 10.2 ± 6.9 days).

### Eating disorder diagnoses and psychiatric co-morbidities:

There was no statistical difference observed in eating disorder diagnoses between SM AYA and heterosexual AYA (Table 2). SM AYA had significantly higher percentages of depression, anxiety, and PTSD compared to heterosexual peers (*p* = 0.003, *p* = 0.008, and *p* < 0.001 respectively) (Table 2). SM AYA were more likely to have a psychiatric co-morbidity (69% versus 48%, *p* < 0.001; RR = 1.46, 95% CI [1.09, 1.93], *p* = 0.009) and more likely to be taking psychiatric medication (RR = 1.56, 95% CI [1.12, 2.21], *p* = 0.012) (Table 3). SM AYA were more likely to have a diagnosis of depression (RR = 1.59, 95% CI [1.10, 2.29], *p* = 0.014), anxiety (RR = 1.57, 95% CI [1.08, 2.29], *p* = 0.019), and PTSD (RR = 4.88, 95% CI [1.94, 12.26], *p* = 0.001) compared to heterosexual peers when adjusted for age, sex, and eating disorder diagnosis (Table 3).

**Table 2:** Eating disorder diagnosis and psychiatric comorbidities for adolescents and young adults hospitalized for complications of malnutrition by sexual orientation^a^

Characteristic	Sexual orientation			
	Total (N = 463)^b^	Heterosexual (N = 364)^c^	Sexual Minority (N = 99)^c^	*p*-Value^d^
*Eating disorder diagnosis*				0.147
Anorexia nervosa^e^	58.10 (269)	60.44 (220)	49.49 (49)	
Other Specified Feeding and Eating Disorder (OSFED)^f^	31.97 (148)	30.22 (110)	38.38 (38)	
Other^g^	9.94 (46)	9.34 (34)	12.12 (12)	
*Presence of other psychiatric diagnosis*^*h*^				** < 0.001**
Yes	52.48 (243)	48.08 (175)	68.69 (68)	
No	47.52 (220)	51.92 (189)	31.31 (31)	
*Specific psychiatric diagnosis*				
Depression^i^	30.24 (140)	26.92 (98)	42.42 (42)	**0.003**
Anxiety^j^	28.73 (133)	25.82 (94)	39.39 (39)	**0.008**
Obsessive–compulsive disorder (OCD)	5.18 (24)	5.22 (19)	5.05 (5)	0.946
Post-traumatic stress disorder (PTSD)	4.10 (19)	2.20 (8)	11.11 (11)	** < 0.001**
Attention deficit hyperactivity disorder (ADHD)	3.02 (14)	3.02 (11)	3.03 (3)	0.997
*Suicide or self-harm*^*k*^				
During hospitalization	7.99 (37)	6.59 (24)	13.13 (13)	**0.033**
Prior to hospitalization	22.25 (103)	18.13 (66)	37.37 (37)	** < 0.001**

Bold face indicates significant p value

^a^Table values are % (N)

^b^Total number of participants with eating disorder diagnosis and sexual orientation listed in chart

^c^Percentages may not sum to 100% due to rounding

^d^T-test was used for continuous variables. Chi Square (or Fisher's exact test if n < 5) was used for categorical variables

^e^Anorexia nervosa includes both restricting subtype and binge/purge subtype

^f^OSFED includes Atypical Anorexia Nervosa and Purging Disorder

^g^Other includes Avoidant Restrictive Food Intake Disorder, Bulimia nervosa, and Unspecified Eating Disorder

^h^Presence of psychiatric diagnosis other than eating disorder

^i^Depression includes major depressive disorder, depressive disorder not otherwise specified, and unspecified depression

^j^Anxiety includes generalized anxiety disorder, social anxiety disorder, anxiety not otherwise specified, and unspecified anxiety

^k^Suicide includes suicide attempt, active suicidal ideation, and passive suicidal ideation

**Table 3:** Associations between psychiatric co-morbidities and sexual orientation^a^

Outcome	Sexual Minority, RR (95% CI)^b^	*p*
Presence of psychiatric co-morbidity	1.46 (1.09, 1.93)	**0.009**
Psychiatric medication use	1.56 (1.12, 2.21)	**0.012**
Depression	1.59 (1.10, 2.29)	**0.014**
Anxiety	1.57 (1.08, 2.29)	**0.019**
Obsessive–compulsive disorder (OCD)	1.01 (0.13, 2.38)	0.980
Post-traumatic stress disorder (PTSD)	4.88 (1.94, 12.26)	**0.001**
Attention deficit hyperactivity disorder (ADHD)	1.14 (0.31, 4.16)	0.844
Suicidality during hospitalization	1.80 (0.90, 3.56)	0.095
History of self-harm or suicidality	1.94 (1.29, 2.92)	**0.001**

Bold face indicates significant *p* value

^a^Abbreviated output of modified Poisson regression analyses adjusted for age, sex assigned at birth, and eating disorder diagnosis

^b^Heterosexual participants are reference category

SM AYA were more likely to have a history of self-injurious behavior or suicidality compared to heterosexual peers (RR = 1.94, 95% CI [1.29, 2.92], *p* = 0.001) (Table 3). There was no difference between groups in active suicidality during hospitalization (Table 3).

## Discussion

Despite the high prevalence of eating disorders among SM AYA, no studies to our knowledge have examined the medical and psychiatric characteristics of this population hospitalized for medical instability. Previous studies have described higher BMIs among SM individuals, specifically those assigned female at birth [32, 33]. Our study demonstrates that SM AYA presented with equally severe vital sign instability despite having a higher %mBMI on admission compared to heterosexual peers. This suggests that higher weight is not protective [34]. This research further highlights the need for medical providers caring for SM AYA to understand that they may be medically unstable at any weight, even one presumed to be in a "normal" or higher range.

Our finding that SM AYA with eating disorders have greater psychiatric comorbidities and higher prior history of suicidality compared to heterosexual peers suggests the need for a psychologic assessment and ongoing mental health support in this population. The higher rates of depression, anxiety, and PTSD in our inpatient population has also been seen in SM AYA seeking treatment in residential and outpatient treatment programs for eating disorders [35]. While this study did not explore causative factors for this increased mental health burden, minority stress theory details the stigma-related stressors associated with higher rates of psychopathology, including eating disorder behavior, in sexual minority individuals [16, 36, 37]. Given that psychiatric comorbidities portend worsened eating disorder outcomes, our findings underscore the importance of psychological support for SM AYA admitted for medical stabilization.

This study is limited by its retrospective and observational nature, which precludes causal inferences. Data was collected from a tertiary care hospital in San Francisco, California and may not be generalizable to other inpatient populations. Pre-existing psychiatric history and suicidality were often collected by self-report, introducing recall bias and heterogeneity into diagnostic reporting. Although we focused on sexual orientation for this analysis, future studies could also assess gender identity, which may influence preoccupation with body weight and appearance in SM AYA [23]. Additionally, our study is limited in examining the impact of socioeconomic factors as we do not have information about income, education, and/or housing available for our participants. Future studies should examine the relationship between socioeconomic status, sexual orientation, and eating disorder behaviors.

Strengths of our study include 8 years of clinical data that was collected by a multi-disciplinary team with expertise in eating disorders including physicians, dietitians, and psychologists. It is noteworthy that over 20% of our study population identified as a sexual minority individual which is greater than the United States population of sexual minority youth at approximately 16% [38].

## Conclusions

SM AYA with eating disorders present with higher %mBMI but are equally medically compromised on inpatient admission for medical stabilization as their heterosexual peers. Additionally, SM AYA have more mental health comorbidity and suicidality. By describing the clinical and psychiatric characteristics of this population, clinicians can better tailor affirming, individualized eating disorder treatment for SM AYA with eating disorders that recognizes their increased mental health burden to ensure equitable health outcomes.

## Data Availability

The data that support the findings of this study are available on request from the corresponding author, AC. The data are not publicly available due to confidentiality restrictions e.g., their containing information that could compromise the privacy of research participants.

## Abbreviations

ADHD: Attention deficit hyperactivity disorder
AN: Anorexia nervosa
ARFID: Avoidant restrictive food intake disorder
AYA: Adolescents and young adults
BMI: Body mass index
BN: Bulimia nervosa
DSM-5: Diagnostic and Statistical Manual of Mental Disorders, Fifth Edition
mBMI: Median body mass index
%mBMI: Percent median body mass index
OCD: Obsessive compulsive disorder
OSFED: Other specified feeding and eating disorder
PTSD: Post-traumatic stress disorder
SM AYA: Sexual minority adolescents and young adults
UCSF: University of California, San Francisco
